# Role of mouse *Wdr13* in placental growth; a genetic evidence for lifetime body weight determination by placenta during development

**DOI:** 10.1038/srep13371

**Published:** 2015-08-26

**Authors:** Vijay Pratap Singh, Jomini Liza Alex, B. Jyothi Lakshmi, S Purnima Sailasree, T. Avinash Raj, Satish Kumar

**Affiliations:** 1 CSIR-Centre for Cellular and Molecular Biology, Hyderabad 500007, India

## Abstract

Placental development is essential for implantation and growth of foetus in the uterus of eutherian mammals. Numerous growth factors are responsible for placental development and cell lineage differentiation. Gene knockout mice have shown role of various genes in the placenta. Here using *Wdr13* knockout mice, we show that this gene is important for proper placental development. *Wdr13,* a X-linked gene, expresses in multiple trophoblast cell types of placenta and the mutant placenta had reduced size after 17.5 *dpc* due to reduction of junctional zone (JZ) and labyrinth zone (LZ). We observed reduction in levels of angiopoietin-2 and *cd44* mRNA in *Wdr13* mutant placenta as compared to that in the wild type. Our findings show that *Wdr13* is required for normal placental development and cell differentiation. *Wdr13* heterozygous female placenta when the mutant allele was of maternal origin showed similar defects as those in case of *Wdr13* null placenta. Using two types of heterozygous females carrying either maternally and paternally derived mutant *Wdr13* allele we provide genetic evidence that development of placenta determines body weight of mice for the entire life.

Placenta is an essential organ for normal embryonic development in uterus. In the mouse uterus, soon after implantation at around 4.5 *days post coitum* (*dpc*) placental development starts. Trophoectoderm surrounding the blastocyst, differentiate into variety of trophoblast cell types. The trophoectoderm in direct contact with inner cell mass (ICM) is known as polar trophoectoderm and other which is not in contact with ICM is known as mural trophoectoderm^1^,^2^,^3^. The mural trophoectoderm differentiate into trophoblast giant cells (TGC), which are polyploid cells involved in separation of maternal decidua from placental layers and have function in early invasion, formation of yolk sack and endocrine signaling. The polar trophoectoderm produces two-cell type of early placenta, namely; the ectoplacental cone (EPC) and the chorion^4^,^5^. The EPC gives three trophoblast lineages: a second wave of TGCs adjacent to the maternal decidua, spongiotrophoblast (SpT) and glycogen trophoblast cells (GCs). Together these cells form the middle junctional zone of placenta. In contrast chorion gives syncytiotrophoblast and other trophoblast cell types of the labyrinth zone (LZ). In the LZ, the allantoic mesoderm gives rise to blood vessels and mesenchyme where as trophoblast cells gives rise to several epithelial derivatives. LZ is the site for foetal-maternal exchange^6^.

All the trophoblast giant cells (TGCs) are polyploid in nature with endocrine activity. However, they differ in their polyploidy, developmental origin, gene expression profiling and location^4^. Cell lineage tracing studies have shown that parietal TGCs (P-TGCs) and canals TGCs (C-TGCs) are originated from both *Tpbpa* positive and negative EPC progenitors. However spiral artery-associated TGCs (SpA-TGCs) are originated from *Tpbpa* positive and sinusoids TGCs (S-TGCs) from *Tpbpa* negative EPC progenitors^4^. However, despite all these differences all TGCs require Hand1 for proper differentiation^7^. The middle layer of placenta (JZ) contains spongiotrophoblast (SpT) and glycogen trophoblast cells (GCs). During the second half of pregnancy some GCs migrate into decidua and crowd around area of spiral arteries^8^. However, there is no evidence of movement of SpT and these remain in JZ. *Tpbpa* is a marker for both SpT and GCs. The origin of GCs is not understood properly. Some studies suggest that GCs originate from SpT while others suggest that GCs may originate directly from progenitors present in EPC^8^,^9^.

*Wdr13* is a member of WD-repeat protein gene family and it expresses in various tissues in mouse and humans^10^,^11^. This gene is present on X- chromosome and is evolutionary conserved through out vertebrate evolution^10^,^11^. Our earlier work showed that *Wdr13* mutant mice have slightly lower body weight at one month of age and become mildly obese with age as compared to Wild type littermates^12^,^13^. However, expression and role of this protein in placenta has not been documented. In the present study, we demonstrate that *Wdr13* is expressed in placenta and has a role in placental development. Further, we took advantage of the preferential inactivation of paternally derived X-chromosome in the female placenta^14^ and show that *Wdr13* heterozygous placenta- when the mutant allele is of maternal origin, have similar defects as in the case of *Wdr13* null placenta (X^*Wdr13*^Y, X^*Wdr13*^X^*Wdr13*^) and provide genetic evidence that placental development affects the lifetime body weight.

## Results

**Figure 1:**
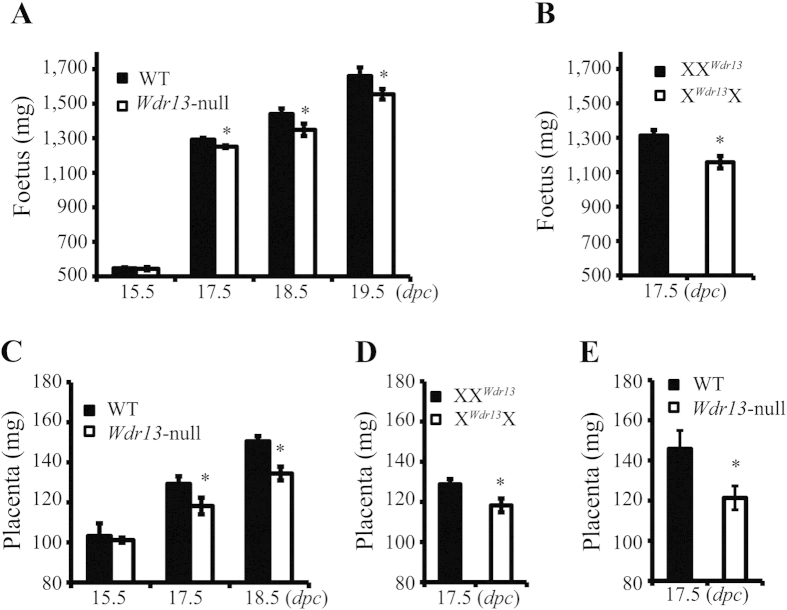
*Wdr13*-null foetus and placenta are smaller than wild type littermate control. (**A**) Weight of *Wdr13*-null (X^*Wdr13*^Y, X^*Wdr13*^X^*Wdr13*^) and wild type (XY, XX) foetus at various time points (n = 35–41). (**B**) Weight of maternally and paternally derived heterozygous females at 17.5 *dpc* (n = 9–11) (X^*Wdr13*^designates an X chromosome carrying *Wdr13* mutation and maternally derived X chromosome is designated first). (**C**) Weight of *Wdr13*-null (X^*Wdr13*^Y, X^*Wdr13*^X^*Wdr13*^) and wild type (XY, XX) placenta at various time points (n = 35–41). (**D**) Weight of maternally and paternally derived heterozygous females placenta at 17.5 *dpc* (n = 9–11). (**E**) Weight of *Wdr13*-null and wild type placenta at 17.5 *dpc* from embryo transferred surrogate mother (n = 7–10).

The first set of experiments were sought to determine the effect of *Wdr13* genotype on the weight of embryos. To generate *Wdr13* null and wild type female and male mice, we crossed *Wdr13* heterozygous female (X^*Wdr13*^X, XX^*Wdr13*^) with either hemizygous male (X^*Wdr13*^Y) or wild type male (XY) [X^*Wdr13*^designates an X chromosome carrying *Wdr13* mutation and maternally derived X chromosome is designated first]. At 19.5 *dpc* the weight of *Wdr13* null mice (X^*Wdr13*^Y, X^*Wdr13*^X^*Wdr13*^) were significantly lower than wild type controls (XY, XX) (Fig. 1A).

The reduction in weight of 19.5 *dpc Wdr13* null mice as compared to wild type mice may either be due to deficiency of *Wdr13* gene felt in placenta or due to deficiency of this gene in embryo or both. To distinguish among these possibilities, weight of *Wdr13* null and their wild type littermate’s embryo/placenta were measured at various time points. At 15.5 *dpc* there was no difference either in placenta or in the embryo weights between these two genotypes (Fig. 1A,C). However, from 17.5 *dpc* onward the weights of both *Wdr13* null placenta and embryos were significantly lower (Fig. 1A,C). These results suggested the role of *Wdr13* in placental development.

**Figure 2:**
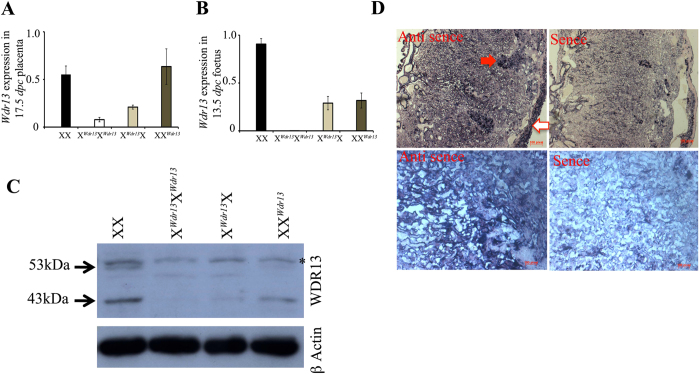
Expression of *Wdr13* in placenta. (**A**) *Wdr13* gene expression in maternally and paternally derived heterozygous females placenta as compared to wild type females at 17.5 *dpc*. (n = 6) (**B**) *Wdr13* gene expression in maternally and paternally derived heterozygous females embryo as compared to wild type females at 13.5 *dpc*. (n = 6) (**C**) Immunoblot for WDR13 protein expression in placenta from different genotype. Lower panel shows beta actin as loading control. *Shows non-specific band. Full-length immunoblots are included in Supplemental Fig. S2. (**D**) RNA *in situ* using *Wdr13* cDNA antisense as probe shows expression in junctional and labyrinth zone of placenta. Sense probe was used as control. Lower panel shows higher magnification image. Filled arrow shows SpT, Closed arrow shows decidua layer.

*Wdr13* gene is present on X-chromosome^10^ and it is known that paternally derived X-chromosome gets preferentially inactivated in mouse placenta^14^,^15^. This presented us an opportunity to create conditional *Wdr13* gene null mutation in placenta only. To achieve this we crossed *Wdr13* heterozygous females with wild type males in order to generate maternally derived heterozygous (X^*Wdr13*^X) placenta/ embryos and hemizygous males were crossed with wild type females to generate paternally derived heterozygous (XX^*Wdr13*^) placenta/embryos. These crosses provided us two types of heterozygous female embryos: those developing in *Wdr13* null placenta due to inactivation of wild type allele on paternal X chromosome and the maternal allele being the mutant, and those developing in heterozygous placenta where mutant allele was present on inactivated paternal chromosome and wild type allele on maternal chromosome was intact. Indeed, qRT-PCR and western blot analysis of placenta and embryo confirmed this duality (Fig. 2). The expression of *Wdr13* in placenta was analyzed by qRT-PCR, using *Wdr13* specific primers (Supplemental Table S1). X^*Wdr13*^X heterozygous placenta had only traces of expression (Fig. 2A) in contrast to XX^*Wdr13*^ heterozygous and wild type placenta. It may be noted that there was no difference in the level of expression of *Wdr13* gene in these two types of heterozygous, X^*Wdr13*^X and XX^*Wdr13*^ embryos (Fig. 2B). Consistent with qRT-PCR, western blot analysis showed expression of both 53 kDa and 43 kDa isoforms of WDR13 protein (Unpublished data) in wild type placenta (Fig. 2C) and as expected in *Wdr13* null placenta both isoforms were absent, confirming authenticity of the antibody. XX^*Wdr13*^ heterozygous placenta showed both isoforms of WDR13 protein as in wild type. However, in X^*Wdr13*^X heterozygous placenta only traces of these isoforms were observed (Fig. 2C).

In the background of above data we measured the weight of placenta/embryo of X^*Wdr13*^X and XX^*Wdr13*^ heterozygous genotype at 17.5 *dpc*. Interestingly weight of placenta and embryo of X^*Wdr13*^X genotype were lower as compared to XX^*Wdr13*^ genotype (Fig. 1B,D). These results show that *Wdr13* has role in placental development and further, lower placental weight contributes to lower body weight of embryo.

To rule out any interaction between uterine genotype and embryo genotype on development of placenta, embryo transfer experiments were performed. 0.5-*dpc* embryos (XY, XX, X^*Wdr13*^Y and X^*Wdr13*^X^*Wdr13*^) were transferred to wild type CD1 pseudo pregnant female. At 17.5 *dpc* pregnant mice were dissected and weight of placenta/embryo was measured. Consistent with our above-mentioned results embryo transferred *Wdr13* null mice placenta showed less weight as compared to wild type littermates (Fig. 1E).

To further see the expression of *Wdr13* gene in various cell types of placenta, RNA *in situ* was performed. RNA *in situ* with *Wdr13* anti sense probe shows expression of *Wdr13* mRNA most prominently in labyrinth zone (Fig. 2D lower panel). A lower level of expression of *Wdr13* mRNA was observed in junctional zone SpT cells but no expression was observed in GCs (Fig. 2D upper panel). Some expression of *Wdr13* mRNA was also observed in decidua layer of placenta (Fig. 2D upper panel).

**Figure 3:**
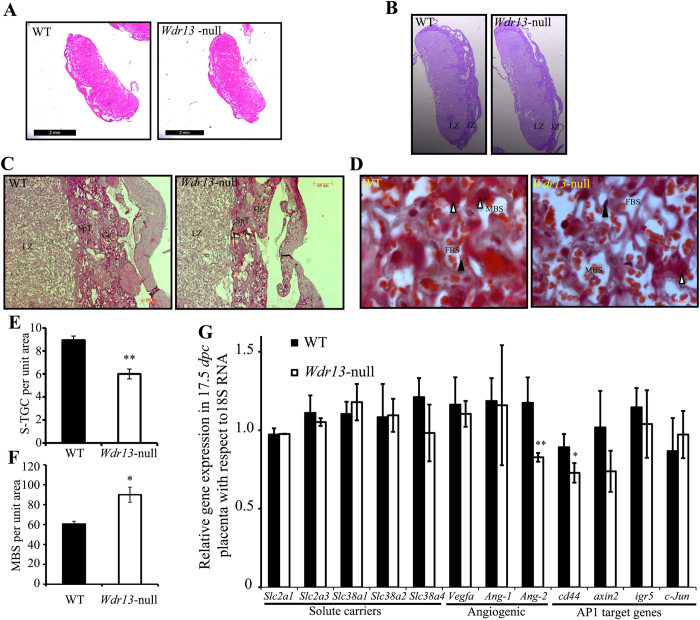
*Wdr13* is important for junctional and labyrinth zone development. (**A**) Gross histology of 17.5 *dpc* placenta (H&E staining) shows reduced placental size in *Wdr13* mutant placenta as compared to wild type. (**B**) Periodic acid-Schiff (PAS) staining of 17.5 *dpc* placenta shows reduced junctional zone in *Wdr13* mutant placenta as compared to wild type. (**C**) Magnified image of PAS staining. (**D**) Masson’s trichrome staining of 17.5 *dpc* placenta shows increased maternal blood space and reduced number of trophoblast giant cells in labyrinth zone of *Wdr13* mutant placenta as compared to wild type. Filled arrowhead shows fetal endothelial cells and closed arrowhead show S-TGC. (**E**) Quantification of number of sinusoidal trophoblast giant cells in labyrinth zone of *Wdr13* mutant and wild type placenta (n = 4). (**F**) Quantification of maternal blood space (MBS) in labyrinth zone of *Wdr13* mutant and wild type placenta (n = 4). (**G**) Expression of various genes involved in glucose and amino acid transport, angiogenesis and differentiation (AP 1 target genes) in 17.5 *dpc Wdr13* mutant and wild type placenta (n = 4–6).

After establishing that WDR13 expresses in placenta and *Wdr13* mutant placenta have reduced weight as compared to wild type placenta, next we performed histological examination to understand the role of this gene in placental development. H&E staining showed overall reduction in placental size of *Wdr13* mutants as compared to wild type placenta (Fig. 3A) due to reduction in junctional zone and labyrinth zone (Table 1). To further see the detailed structure of placental junctional zone, Periodic acid-Schiff (PAS) staining was performed for staining GCs of JZ at 17.5 *dpc*. PAS staining showed over all decrease in junctional zone SpT and GCs (Fig. 3B,C) in *Wdr13* null placenta as compared to wild type placenta. For detailed structure of placental labyrinth zone, Masson’s trichrome (MT) staining was performed at 17.5 *dpc*. MT staining showed significant reduction in the number of S-TGCs in *Wdr13* mutant placenta as compared to that in wild type placenta (Fig. 3D,E). Interestingly, we observed increased maternal blood space in *Wdr13* mutant as compared to wild type placenta (Fig. 3D,F) probably as compensatory mechanism to increase the nutrient supply.

At least three possibilities exist for the reduction in the number of various trophoblast cells in *Wdr13* mutant placenta: 1) reduced cell proliferation, 2) increased apoptosis and 3) affected EPC, chorion differentiation. So we performed cell proliferation assay in placenta by injecting BrdU in pregnant female mice. At 17.5 *dpc* there was no difference in number of BrdU positive in *Wdr13* mutant and wild type placenta (Supplemental Fig. S1A). Next we performed apoptosis using TUNEL assay on placenta sections. At 15.5 *dpc* number of apoptotic cells were similar in both *Wdr13* mutant and wild type placenta (Supplemental Fig. S1B). It appears that reduction in the number of various trophoblast cells in *Wdr13* mutant placenta may be due to differentiation defects in EPC, chorion and their progenitors. So we performed gene expression analysis for gene(s) involved in solute transport, angiogenesis and differentiation. There was no difference in glucose and amino acid transporter in *Wdr13* mutant and wild type placenta at 17.5 *dpc* (Fig. 3G). Interestingly, angiogenic gene angiopoietin-2 was significantly down regulated in *Wdr13* mutant as compared to wild type placenta (Fig. 3G) where as there was no change in angiopoietin-1 and vascular endothelial growth factor (VEGF). Parallel study in our lab showed that *Wdr13* activate AP1 target genes in presence of JNK signaling (Unpublished data). So we analyzed some AP1 target genes having role in placental trophoblast cell differentiation. We observed that *cd44* was down regulated in *Wdr13* null placenta as compared to wild type placenta whereas there was no change in *axin2*, *igr5* and *c-Jun* levels.

**Figure 4:**
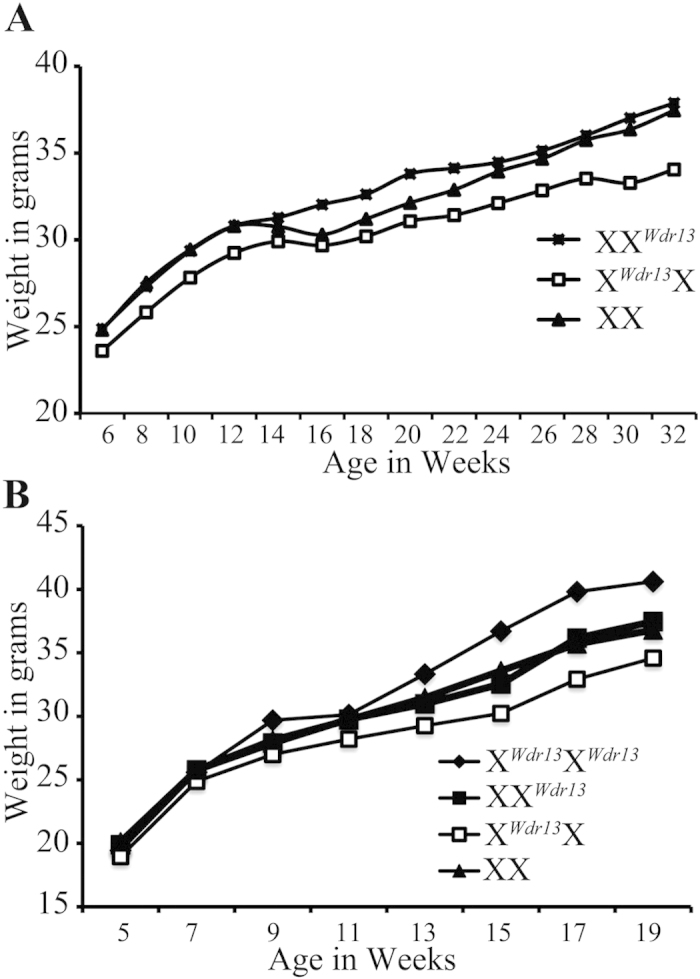
Growth curve of maternally and paternally derived *Wdr13* heterozygous female as compared to wild type females after birth on chow (A) and high fat diet (B).

The next set of experiments were sought to determine the effect of *Wdr13* null placenta phenotype on postnatal growth of mice. As shown above that X^*Wdr13*^X heterozygous placenta was deficient in WDR13 protein and XX^*Wdr13*^ heterozygous placenta showed WDR13 protein levels as comparable to wild type while the two heterozygous female embryos (X^*Wdr13*^X and XX^*Wdr13*^) had comparable levels of *Wdr13* expression (Fig. 2B). We measured the body weight of XX, X^*Wdr13*^X and XX^*Wdr13*^ females on normal chow as well as on high fat diet and found that on both diets regimes XX and XX^*Wdr13*^ females showed higher body weight as compared to X^*Wdr13*^X females from birth till termination of experiments (Fig. 4A,B). These results indicate that due to the impaired placental development in X^*Wdr13*^X females their body weight is lower throughout their life suggesting that gene expression set during early development influenced their body weights during adulthood. This is a strong genetic evidence to support that during initial development placenta determines body weight for entire life.

## Discussion

Numerous growth factors and genes have been implicated in placental growth, function and development^16^,^17^,^18^. Role of various WD repeat protein such as *Fbxw8* and RACK1 have been shown in placental development^19^,^20^. In the present study, we show for the first time that *Wdr13* gene, which is a member of WD repeat protein, expresses in placenta and the lack of this protein causes reduction in size of placenta. The reduction in placental size is mainly due to reduction in JZ and LZ (Table 1). Consequently, this reduction in size of placenta causes reduced body weight of embryo after birth.

*Wdr13* gene expresses in placenta in decidua, SpT of JZ and in LZ. The reduction in the number of various trophoblast cell type indicates role of *Wdr13* gene during the development of placenta. These trophoblast cells are derived from EPC and chorion^4^. P-TGCs, S-TGCs, and syncytiotrophoblast cells originate from *Tpbpa*^*−*^ cells whereas SpT originates from *Tpbpa*^*+*^ cells of EPC and chorion^4^. So it is unlikely that *Wdr13* has specific role in particular cell type. At present we do not know the expression of *Wdr13* in EPC, chorion; and *Wdr13* mutant placenta shows weight difference only after 15.5 *dpc*. So it is likely that *Wdr13* has role in these cell types only in late stages of development. To further understand the cell type specific expression of *Wdr13* in placenta requires experimentations using cell type specific markers.

Placental angiogenesis is regulated by vascular endothelial growth factor (VEGF) and its high affinity receptor tyrosine kinases VEGFR-1, VEGFR-2 along with angiopoietin-1 (Ang-1), angiopoietin-2 (Ang-2) and its receptor Tunicainterna endothelial cell kinase-2 (Tie-2)^21^,^22^,^23^. VEGF, Ang-1 and Ang-2 are expressed in cytotrophoblast, syncytiotrophoblast and endothelial cells with in placenta^24^,^25^. VEGF and Ang-1 promote angiogenesis whereas Ang-2 antagonize Ang-1 function in endothelial cells^26^. In trophoblast cells Ang-2 and to a lesser extent Ang-1 promote DNA synthesis and cell growth^21^. In *Wdr13* knockout placenta we observed a reduction in Ang-2 mRNA levels as compared to that in wild type placenta, while the levels of VEGF and Ang-1 were comparable between these two genotypes (Fig. 3G). Reduced level of Ang-2 mRNA may be one of the contributing factors in reduction of placental growth of *Wdr13* mutant mice. In the case of human placenta, down regulation of Ang-2 is associated with intrauterine growth restriction of placenta^21^. However, at this time we do not know the reduction of Ang-2 mRNA is in endothelial cells or trophoblast cells, which requires further experimentation.

Ang-2 promoter contains AP-1 sites^27^ and parallel studies in our lab have shown that WDR13 activates AP1 target genes in presence of JNK signaling by interacting with c-Jun (Unpublished data). Thus, it is possible that down regulation of Ang-2 gene is directly regulated by WDR13. In support of these results analysis of gene expression of various AP1 target genes showed reduced expression of *cd44* in *Wdr13* knockout placenta as compared to wild type placenta (Fig. 3G). *cd44* gene expresses in placenta and is important for cell migration and differentiation^28^,^29^. Various AP1 components like JunB and Fra1 are essential for placenta formation particularly in LZ^30^,^31^. So it appears that reduced expression of AP1 target genes may responsible for reduced placenta weight in *Wdr13* mutant mice.

The relation between placental size and body weight of young one is well documented and lower birth weight is associated with metabolic syndrome including type 2 diabetes, insulin resistance, dyslipidemia and non-alcoholic fatty liver disease^32^,^33^. Due to preferential inactivation of paternal X-chromosome X^*Wdr13*^X placenta shows characteristics *Wdr13* null placenta where as XX^*Wdr13*^ placenta shows characteristics of wild type placenta. Due to different characteristics of these two types of placenta, X^*Wdr13*^X heterozygous female shows less body weight as compared to XX^*Wdr13*^ females. Interestingly, both X^*Wdr13*^X and XX^*Wdr13*^ heterozygous females have same genetic contents and similar levels of *Wdr13* expression (Fig. 2B) but they differ only in placental environment. What is the impact of reduced body weight in later stages of life and how will these two heterozygous females develop in wild type placental environment is not understood at present? Detailed metabolic parameter analysis and tetraploid complementation experiments in order to maintain a wild type placental environment for these two types of heterozygote females will be needed to understand these questions.

In summary, we provide evidence for role of *Wdr13* gene in mouse placenta. *Wdr13* gene expresses in placenta and *Wdr13* mutant placenta shows reduced size and reduced number of S-TGC in LZ. The reduction in placental size results in reduced embryo weight. We provide genetic evidence to support that initial placental development determines the body weight for the entire life.

## Methods

### Animal experimentation

All the mice experiments were approved by and performed under guidelines of the institutional animal ethics committee of CSIR-Centre for Cellular and Molecular Biology, Hyderabad, India. All methods were carried out in accordance with the approved guidelines. *Wdr13* mutant mice on CD1 genetic background were genotyped by PCR as described previously using primers mentioned in Supplemental Table S1^12^. *Wdr13* heterozygous females were crossed with wild type male to generate wild type, knockout and maternally derived heterozygous (X^*Wdr13*^X) placenta and embryos. Hemizygous males were crossed with wild type females to generate paternally derived heterozygous (XX^*Wdr13*^) placenta and embryos. At given time point of pregnancy female mice were dissected and weight of individual placenta and embryos were recorded. Genotyping of individual placenta and embryo was confirmed by isolating DNA from embryo tail as described previously^12^. The gender of individual placenta and embryo was confirmed by using *Sry* primers (Supplemental Table S1). Maternally derived heterozygous (X^*Wdr13*^X), paternally derived heterozygous (XX^*Wdr13*^) and wild type females body weight was measured fortnightly on normal diet and on high fat diet.

### Uterine transfer of mouse embryos

Isolation and transfer of mouse embryo in oviduct were performed as described previously^34^. Briefly, fertilized eggs were isolated from oviduct of pregnant mice at 0.5 *dpc* and incubated in M16 medium. Same day embryos were transferred to oviduct of pseudo-pregnant female (CD1) after mating with vasectomized male.

### Histochemistry, *in situ* hybridization, cell proliferation and apoptotic assay

Placentas were collected in PBS, fixed in 4% paraformaldehyde overnight and processed as described previously^12^. Briefly, 4 μm thick paraffin embedded sections were made and mounted on positively charged slides (Fischer scientific). Slides were stained with Hematoxylin-Eosin (Sigma) for visualizing tissue morphology by light microscopy. Periodic acid-Schiff (Sigma) and Masson’s trichrome (Sigma) staining were performed using standard procedure. For RNA *in situ* of *Wdr13* gene in placenta, primer pairs 5′-aacgcctaccgtacaccaac-3′ and 5′-acatggtacatgcctgcaaa-3′ were used to generate the anti sense and sense probe. Paraffin embedded placenta sections were used to perform RNA *in situ* using DIG RNA labeling kit from Roche applied sciences (cat no-11175025910) as per manufacturers instructions. To assay the cell proliferation in placenta pregnant female mice were injected BrdU (100 mg/kg body weight), sacrificed 1.5h after injection and fixed and processed as described above. BrdU staining was performed using Anti-BrdU Antibody (Sigma) followed by staining with anti mouse HRP conjugated secondary antibody and developed with DAB substrate (Cat no-11718096001, Roche). The number of positive cells were counted manually using Axioskop (Axivision software). TUNEL assays were performed using Dead END Fluorometric TUNEL System (Promega). Images were recorded in confocal microscope and TUNEL-positive cells were counted manually from the images from total of 4 mice per genotype.

### Data quantification and statistical analysis

Cavalieri technique (Simple grid method) was used to measure area of placenta, JZ, LZ and MBS^2^,^35^. Total placenta area and area of various placental layer were quantified using ImageJ software (NIH; http://rsb. info.nih.gov/ij/) on H&E stained samples. A minimum of four placenta for each genotype and at least three sections from each placenta was used to quantify placental area. For counting S-TGCs in labyrinth layer Masson’s trichrome stained placenta were imaged with 1000X magnification. At least four placentas from each genotype were analyzed with three to four field from each placenta. For MBS Masson’s trichrome stained placenta were imaged and ImageJ software was used to calculate MBS per unit area. MBS was calculated from minimum of four placentas per genotype with three to four field per placenta. For determining statistical significance an unpaired two-tailed student’s *t*-test with unequal variance was used to compare genotypes.

### RNA isolation, real time PCR and western blot analysis

Total RNA from 17.5 *dpc* placenta was isolated using RNeasy Mini Kit (Qiagen) and reverse transcription was performed using ImProm-IITM kit (Promega) after DNase (Promega) treatment of RNA samples. Real time PCR was performed for various genes using Syber Green master mix (Invitrogen) and list of primers sequences is provided in Supplemental Table S1. For western blot analysis, 17.5-*dpc* placentas were lysed in RIPA buffer, quantified and blotted on PVDF membrane. Anti-WDR13 purified antibody (HPA000913) from Sigma and beta actin (sc-47778) from Santacruz were used for visualization of the protein.

## Additional Information

**How to cite this article**: Singh, V. P. *et al.* Role of mouse *Wdr13* in placental growth; a genetic evidence for lifetime body weight determination by placenta during development. *Sci. Rep.* **5**, 13371; doi: 10.1038/srep13371 (2015).

## Supplementary Material

Supplementary Information

## Figures and Tables

**Table 1:** Morphometric analysis of *Wdr13* null mice placenta with respect to wild type placenta on day 17.5 p.c. *

Location	Cross section area (mm^2^)		*P*
	Wild type	*Wdr13*-null	
Total placenta	9.83 ± 0.42	8.40 ± 0.34	0.016
Labyrinth layer	6.79 ± 0.34	6.02 ± 0.22	0.055
Junctional layer	2.85 ± 0.24	2.22 ± 0.15	0.047

^*^Quantification of total placental area and placental layers was performed using ImageJ software on at least four placentas for each genotype and at least three sections from each placenta. For determining statistical significance an unpaired two-tailed student’s *t*-test with unequal variance was used to compare genotypes.
