# Supplementary material for: Role of mouse Wdr13 in placental growth; a genetic evidence for lifetime body weight determination by placenta during development
Source: Sci Rep. 2015 Aug 26;5:13371. doi: 10.1038/srep13371 (PMC4549788; doi:10.1038/srep13371)
Supplement: Supplementary Information [file srep13371-s1.pdf]

Role of mouse *Wdr13* in placental growth; a genetic evidence for lifetime body weight determination by placenta during development

Vijay Pratap Singh, Jomini Liza Alex, B Jyothi Lakshmi, S Purnima Sailasree, T Avinash Raj and Satish Kumar

Supplemental Table S1: Primer list used for PCR
Slc2a1 FP: 5'-CCTATGGCCAAGGACACACT-3'<br>*Slc2a1* RP: 5'-CTGGTCTCAGGCAAGGAAAG-3'
Slc2a3 FP: 5'-GCAATGGAAAGGTGTGAGGT-3'<br>*Slc2a3* RP: 5'-AAGTGGAGGAGGGGACAGTT-3'
Slc38a1 FP: 5'-ATTTGAGCTGGCCAAGAAGA-3'<br>*Slc38a1* RP: 5'-CGTCCTGGTTCGTGATTTTT-3'
Slc38a2 FP: 5'-CGCTGCTCTCTTTGGATACC-3'<br>*Slc38a2* RP: 5'-GAGTGACGGAACTCCGGATA-3'
Slc38a4 FP: CCTTGGCTACACCAGTGGAT-3'<br>*Slc38a4* RP: TTAGGCAGCGAGATCATGTG-3'
Vegfa FP: 5'-CAGGCTGCTGTAACGATGAA-3'<br>Vegfa RP: 5'-GCATTCACATCTGCTGTGCT-3'
Ang-1 FP: 5'-ATGGTTTGCCTGTCCACTTC-3'<br>*Ang-1* RP: 5'-GGAACCAGTGTTCTGGGCTA-3'
Ang-2 FP: 5'-AGCCCAGGTCCTTTGTTCTT-3'<br>*Ang-2* RP: 5'-GCTTGGCATCATAGTGCTGA-3'
cd44 FP: 5'- CTCCTGGCACTGGCTCTGA-3'<br>*cd44* RP: 5'-CTGCCCACACCTTCTCCTACTATT-3'
axin2 FP: 5'-GGTTCCGGCTATGTCTTTGC-3'<br>*axin*2 RP: 5'-CAGTGCGTCGCTGGATAACTC-3'
gpr49 FP: 5'-CGGAGGAAGCGCTACAGAAT-3'<br>*gpr49* RP: 5'-CTGGGTGGCACGTAGCTGAT-3'
c-Jun FP: 5'- CTGATCATCCAGTCCAGCAA-3'<br>*c-Jun* RP: 5'-GACACTGGGAAGCGTGTTCT-3'
18S FP: 5'-GCTTAATTTGACTCAACACGGGA-3'<br>*18S* RP: 5'-AGCTATCAATCTGTCAATCCTGTC-3'
gapdh FP: 5'-ACCCAGAAGACTGTGGATGG-3'<br>*gapdh* RP: 5'-CACATTGGGGGTAGGAACAC-3'
Wdr13 FP: 5'-GTACGGGCCACTCTCAGAAC-3'<br>*Wdr13* RP: 5'-CTGCAGCTGGTAGGAACCTC-3'
Sry FP- 5'-CCCAGCATGCAAAATACAGA -3'<br>*Sry* RP- 5'-AACAGGCTGCCAATAAAAGC -3'
Wdr13 Genotyping<br>*Wdr13*E1F- 5'-CCAACAGAGCTGCAAATTGA-3'<br>*Wdr13*E2R- 5'-TGCTATAGGCACGAGCACTG-3'<br>*Wdr13* NeoRP- 5'-AATTCGCCAATGACAAGACG-3'

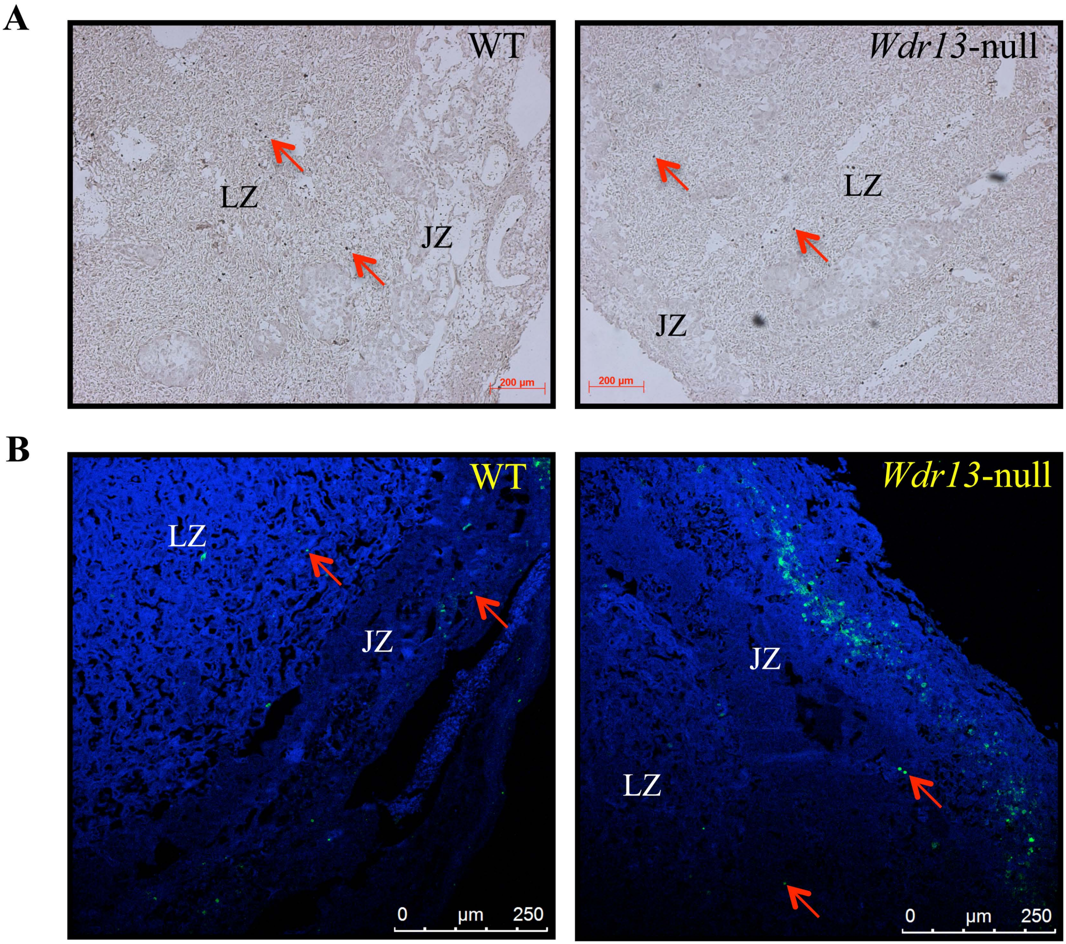

Supplemental Figure S1 │ Cell proliferation (A) by counting the number of BrdU positive cells (17.5 *dpc*) and apoptosis (B) by TUNEL assay (15.5 *dpc*) in *Wdr13*-null and wild type littermate control placenta.

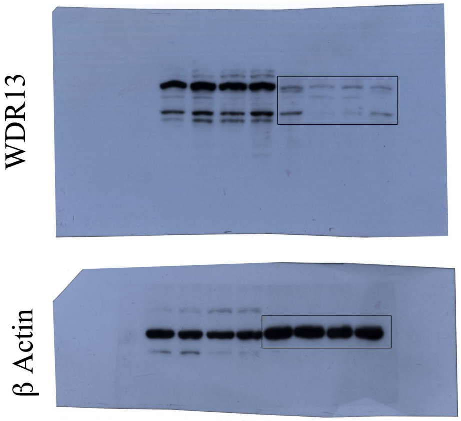

Supplemental Figure S2 | Full length immunoblot for Figure 2C.
